# Identification and characterization of peptide: N- glycanase from *Dictyostelium discoideum*

**DOI:** 10.1186/1471-2091-13-9

**Published:** 2012-06-08

**Authors:** Anuradha Gosain, Rakhee Lohia, Anju Shrivastava, Shweta Saran

**Affiliations:** 1 School of Life Sciences, Jawaharlal Nehru University, New Delhi 110 067, India; 2 Department of Zoology, University of Delhi, New Delhi 110 007, India; 3 Department of Genetics, University of Delhi, South Campus, New Delhi 110 021, India

## Abstract

**Background:**

Peptide: N- glycanase (PNGase) enzyme cleaves oligosaccharides from the misfolded glycoproteins and prepares them for degradation. This enzyme plays a role in the endoplasmic reticulum associated degradation (ERAD) pathway in yeast and mice but its biological importance and role in multicellular development remain largely unknown.

**Results:**

In this study, the PNGase from the cellular slime mold, *Dictyostelium discoideum* (*Dd*PNGase) was identified based on the presence of a common TG (transglutaminase) core domain and its sequence homology with the known PNGases. The domain architecture and the sequence comparison validated the presence of probable functional domains in *Dd*PNGase. The tertiary structure matched with the mouse PNGase. Here we show that *Dd*PNGase is an essential protein, required for aggregation during multicellular development and a knockout strain of it results in small sized aggregates, all of which did not form fruiting bodies. The *in situ* hybridization and RT-PCR results show higher level of expression during the aggregate stage. The expression gets restricted to the prestalk region during later developmental stages. *Dd*PNGase is a functional peptide:N-glycanase enzyme possessing deglycosylation activity, but does not possess any significant transamidation activity.

**Conclusions:**

We have identified and characterized a novel PNGase from *D. discoideum* and confirmed its deglycosylation activity. The results emphasize the importance of PNGase in aggregation during multicellular development of this organism.

## Background

Peptide: N-glycanase (PNGase; EC 3.5.1.52) is a member of transglutaminase (TG) -like superfamily and is characterized by TG -like core domain (called PNGase core domain) with the conserved cysteine (C), histidine (H) and aspartate (D) core residues, similar to the TG enzymes [1]. PNGase catalyzes a deglycosylation reaction and cleaves at β-aspartyl glucosylamine bond and removes complete glycan moiety from the glycoprotein substrate. Its reaction is different from TG catalyzed transamidating or amide bond formation reaction [2]. Mutagenesis studies have confirmed that the catalytic triad residues (C, H, and D) of PNGase are essential for its deglycosylation activity [3].

PNGases are ubiquitously present from prokaryotes to eukaryotes and from lower to higher metazoans*.* The PNGase activity was firstly identified and characterized in almond [4,5] and was called PNGaseA. PNGase from bacteria *Flavobacterium meningosepticum* (PNGaseF) was characterized and found to be similar to PNGaseA as both could deglycosylate high mannose and complex type native glycoproteins [6] but were later reported to be kinetically different in substrate specificity [7]. The first evidence of PNGase in animal was obtained from medaka fish (*Oryzias latipes)* embryo [8]. Two PNGaseMs were characterized having different optimum pH for activity- acidic PNGaseM (3.5–4 pH) and neutral PNGaseM (neutral pH). The expression of these two PNGases was developmentally regulated with neutral PNGase expressing at 4–8 cells stage and early gastrula while acidic PNGaseM expressing after gastrulation [9]. These are responsible for the deglycosylation of various yolk glycoproteins leading to accumulation of *N*- glycans. Subsequently, PNGases were reported in the cytoplasm of mammalian cells [10], hen oviduct [11], *S. cerevisiae*[12] and *Drosophila*[13]. The cytoplasmic PNGases are very different from those found in bacteria and acidic PNGases, as the former require a thiol reactive group, a neutral pH, reducing environment (DTT) for optimum activity and possess unique carbohydrate binding properties [14]. Unlike bacterial PNGase, cytoplasmic PNGase cannot act on native glycoproteins and selectively deglycosylate denatured (non-native) glycoproteins [15]. Subcellular localization studies demonstrated that PNGases are mainly present in the cytoplasm, but there are reports of its membrane association, especially with the endoplasmic reticulum membrane [11] and its presence in nucleus, in case of *S. cerevisiae *[16] has been confirmed. PNGase activity was found to be growth phase dependent in *S. cerevisiae* with no activity in logarithmic phase cells and high activity during stationary phase cells [12]. *S. cerevisiae png1p*^*-*^ strain is not affected in growth rate and viability, so it does not seem to be an essential gene in *S. cerevisiae*, but it leads to the accumulation of misfolded glycoprotein in cytosol and a delay in its degradation [16]. The *C. elegans png1p*^*-*^ mutants are defective in axon development and branching [17]. Recently, Maerz *et al *[18] have shown that the PNGase from filamentous ascomycete, *Neurospora crassa*, lack deglycosylation activity but is essential for cell polarity as well as for cell wall integrity and DNA repair.

Despite accumulating knowledge about PNGases from yeast to mammals, there is not much knowledge about the biological significance of this protein during development and differentiation of organisms. In the present study we have identified and characterized the PNGase from *Dictyostelium discoideum*, a model system having great significance due to the phylogenetic position occupied by it. In the present work, we identified a novel PNGase from *D. discoideum* genome using *in silico* analyses and characterized its role in growth, development and differentiation. The domain architecture and the sequence comparison validated the presence of probable functional domains in *Dd*PNGase. The tertiary structure matched most with the mouse PNGase.

*Dd*PNGase is a functional peptide:N-glycanase enzyme possessing deglycosylation activity, but does not possess transamidation activity. RT-PCR data revealed almost 3–4 fold increase of PNGase transcript during aggregation stage after which they declined suggesting its importance during the process of aggregation. *In situ* hybridization studies revealed the abundance of *png* transcript in the prestalk region, specifically in the pstB and pstAB regions. Our observations suggest that PNGase plays a role in regulating the number of aggregates that develop to form multicellular structures. The *png*^-^ cells make smaller aggregates upon starvation but only few of them proceeded with further development. One could say that *Dd*PNGase is required for the signaling taking place during aggregation whose consequences are required for cell type differentiation mainly of the prestalk cells.

## Methods

### Cell culture, growth and development

*D. discoideum,* Ax2 (axenic strain) cells (unicellular) were maintained in liquid suspension in standard HL-5 medium (pH 6.5) at 22°C with 200 rpm shaking. The medium was supplemented with antibiotics as indicated. Log phase cells at a density of ~2.5–3.5 x 10^6^ cells/ml was used for all the experiments.

Cells were grown at a density of 0.5–1.0 x 10^6^ cells/ml with indicated antibiotics in a flask under shaken conditions and growth was monitored at the indicated times as shown.

To induce multicellular development, cells were harvested from exponentially growing cultures, washed twice in ice cold KK_2_ buffer (16 mMKH_2_PO_4_, 4 mM K_2_HPO_4_.3H_2_O, pH 6.2) and plated at a density of 1.0x10^7^ cells/cm^2^ on non-nutrient agar (1.5% in KK_2_ buffer) plates.

Development was synchronized by incubating the plates with the cells at 4°C for 4–5 hours and then transferring them to 22°C for further development [19].

### In-silico analyses of putative PNGase

The genomic DNA, cDNA and protein sequences of the putative PNGase were obtained from dictybase online resource (http://www.dictybase.org). The protein sequences of known PNGase were obtained from UniProt KB database (http://www.uniprot.org). The domain architectures of the proteins were deduced by Simple Modular Architecture Research Tool, SMART (http://SMART.embl-heidelberg.de). PNGase orthologues were searched by Basic Local Alignment Search Tool (BLASTp) at NCBI (http://blast.ncbi.nlm.nih.gov/Blast.cgi), UniProt and dictybase. Multiple alignments were performed using ClustalW2 at EBI server (http://www.ebi.ac.uk/Tools/clustalw2/). PHYLIP package (Phylogeny Inference Package, version 3.68), was used to construct Neighbor joining (NJ) phylogenetic trees with 1000 bootstrap replicates to create a consensus tree.

### Tertiary structure modeling

Secondary structure of the candidate protein was predicted by Jpred3 (http://www.compbio.dundee.ac.uk/www-jpred/). The protein sequence of putative *Dd*PNGase was used as query in Robetta server (http://robetta.bakerlab.org) for domain prediction which identified proteins of known tertiary structures having significant homology to the primary sequence of the query domains. The best homologous mouse PNGase protein structure (PDB- 2f4mA) was used as a template for comparative modeling of the tertiary structure of the query protein domain using SWISS MODEL server (http://swissmodel.expasy.org/) in alignment mode [20]. The 3D model for *Dd*PNGase was analyzed based upon the crystal structures of *Sc*PNGase [21] and mouse PNGase [22]. The stereochemical quality of the protein model was analyzed through residue-by-residue geometry and overall structure geometry in Ramachandran plot that was obtained with PROCHECK tool at Structure Analysis and VErification Server, SAVES (http://nihserver.mbi.ucla.edu/SAVES/). The superimposed images of the template (mouse PNGase) and *Dd*PNGase tertiary structure was obtained from SuperPose, Version 1.0 (http://wishart.biology.ualberta.ca/SuperPose/) to mark the differences between the two structures.

### RNA detection by *in situ* hybridization and RT-PCR

#### *In situ* hybridization

Cells were incubated at 0.5–1.0 x 10^7^ cells/cm^2^ on dialysis membrane, supported by KK_2_ agar, until the desired developmental stages were attained. *In situ* hybridization with 200 ng/ml of digoxigenin (DIG) labeled *pngase* RNA was carried as described earlier by Escalante and Sastre [23]. A sense *pngase* probe was used as an internal control. The spatial expression pattern of the transcript of *Ddpngase* was investigated using probes obtained by *in vitro* transcription of the exonic region after cloning in commercially available pBSII SK + (pBluescript II phagemid) vector. RNA of 1.2Kb (705 to 1955 nucleotides) was synthesized from 1.2 Kb exonic region amplified by PCR using the primers FP (BamHI): 5′- GATGCGGATCCCTACTATCAACTAAATGTGGAAGATGTGGT-3′ and RP (XhoI): 5′ ACTACTCGAGAATAACCAATTGAAAAACCTGAAATTATATGAG-3′ and digested with BamHI/XhoI and cloned directly into BamHI/XhoI site of pBSII SK+. The construct pBSII SK + (*pngase probe*) was digested with BamHI to yield template for antisense probe synthesis by T7 RNA polymerase, while digestion with XhoI yielded template for sense probe synthesis by T3 RNA polymerase. Sense and antisense probes were processed for hydrolysis due to their larger size (1.2 Kb) using 1x carbonate buffer and incubated at 65°C for 15 min.

#### RT-PCR

For semi-quantitative detection of the transcript, RNA was isolated from cells collected at two hour time intervals from cells developing synchronously on a non- nutrient agar plate using the Trizol reagent (Sigma, USA). RNA was treated with RNase-free DNase and purified using the RNeasy minikit. cDNA was synthesized from 1 μg of total RNA with Thermo script RT-PCR system from Invitrogen using oligo dT primers. RT-PCR reactions were performed using specific primer sets as shown below and *rnl*A was used as an internal control. The cycle numbers used for amplification were within the linear range of amplification. Primer set for the amplification of *pngase* was FP-5′- AATCAATCTAAAACTATAAATTTAATATATAATG-3′ and RP-5′-ATTCTTAAACCAATCCAATAACAT-3′. The primer set used for *rnl*A was FP-5′- GGATTCTGCAAAATGGCAAC-3′ and RP- 5′-GTCCTCTCGTACTAAAGGAAGG-3′.

### Plasmid construction

The *Ddpngase* gene identified by *in silico* analyses was PCR amplified using FP: (BamHI):5′- ACGCGGATCCAATCAATCTAAAACTATAAATTTAATATATAATG-3′and RP: (XhoI):5′- ACTACTCGAGAATAACCAATTGAAAAACCTGAAATTATATGAG-3′ from the genomic DNA and expressed as a fusion protein with the enhanced yellow fluorescent protein (EYFP) at the C-terminal [19]. The expression was driven by the constitutive promoter*, actin 15*. The construct was introduced into wild type Ax2 cells by electroporation and the transformants were selected with 100 μg/ml G418 and was called *act15*/*png-eyfp*/Ax2 strain.

To prepare a *pngase* gene disruption construct, two DNA fragments of the *png* gene comprising nucleotides from 4–518 and from 705–1493 were amplified by PCR from the vector p*act15*/*png- eyfp* using oligonucleotides (first fragment, FP- BamHI: 5′-GACGCGGATCCAATCAATCTAAAACTATAAATTTAATATATAATG-3′ and RP-EcoRI: 5′- CTAGGAATTCATTCTTAAACCAATCCAATAACAT-3′; second fragment, FP-BamHI: 5′-GATGCGGATCCCTACTATCAACTAAATGTGGAAGATGTGGT-3′ and RP-XbaI:5′- CTATCTAGAGCCATACCATCAGCACCATTAGT-3′) that add a 5’-BamHI and 3’-EcoRI site to the first fragment and a 5’- XbaI and 3’-BamHI site to the second fragment. These fragments were three-point ligated with XbaI and EcoRI digested pBsrΔBamH1 vector [24]. The construct was linearized with BamHI which yielded a pBsrΔBamHI plasmid flanked with a 515 and 531 bp of 5’ and 3’ *pngase* sequence respectively and introduced into wild type Ax2 cells. Transformed cells were selected for growth at 10 μg/ml blasticidin and selected clones were screened for homologous recombination by two separate PCR reactions and confirmed by sequencing. The knock outs were called *png*^*-*^/Ax2 strain.

### Deglycosylation assay

Lysates prepared from *act15*/*png-eyfp*/Ax2 and *png*^*-*^/Ax2 strains and the commercially available PNGase F (Sigma Aldrich) were used for comparing the capability to deglycosylate native and denatured form of chicken ovalbumin as per the protocol of Joshi *et al*[25] with minor modifications. Briefly, the lysates (prepared from 1x10^5^ cells) of the different strains were incubated with 80 μg ovalbumin (native or denatured) in 1000 μl reaction containing 5 mM DTT in 20 mM Na_2_HPO_4_ and 0.5 M NaCl at pH 7.2 at 22°C for different time points (0 h-overnight). The reaction was stopped by boiling in SDS sample buffer at 100°C for 5 min and analyzed by Western hybridization. Samples (10 μl) of deglycosylated reaction was resolved on 10% SDS- PAGE and transferred to the nitrocellulose membrane and blocked with 4% non-fat dry milk. The membrane was treated with 1:4000 diluted rabbit polyclonal anti- chicken egg albumin antibody (Sigma Aldrich) in blocking solution, followed by washing with 1x TBST buffer, followed by treatment with 1:6000 diluted goat anti- rabbit IgG-HRP (Bangalore Genei, India) conjugated, in blocking solution. The blot was treated with SuperSignal West Pico chemiluminiscent substrate (Thermo Scientific) and exposed to X-ray film. Film was developed and fixed, and the ovalbumin bands were visualized.

### Transglutaminase activity

Freshly grown log phase cultures of the transformants and the wild type strain were taken for the experiments. The assays were performed using TG assay kit (CS1070, Sigma-Aldrich) according to the manufacturer’s instructions. Briefly, 1.5–2 x 10^7^ cells were lysed to prepare a crude protein extract. Around 10 μg total protein extract was added to each poly-L-lysine coated microplate wells. 0.1 mU pure guinea pig liver TG2 was used as a positive control while the negative control wells did not contain any protein. Assay buffer containing biotin labeled TBQQEL-OH glutamine substrate and DTT was added in equal volumes to the proteins and the reaction was allowed to proceed at 22°C for 60 min. The wells were washed 3 times with MQ water and incubated with 0.1 μ g Streptavidin-Peroxidase in 100 μl PBS-T for 20 min at 22°C. The wells were washed thrice with 200 μl PBS-T without incubation. 200 μl 3,3’,5,5’-tetramethylbenzidine (TMB) liquid substrate was added to each well for 3 min. 100 μl stop solution was added to stop the color development. The absorption was measured at 450 nm. The protein content of each sample was measured by Bradford method using BSA for standards [26]. The results were expressed as percent activity, taking wild type activity as 100%. A minimum of 4–5 independent experiments in triplicates was carried out.

## Results and discussions

Recent evidences in various organisms have established the role of PNGase in ERAD related events but its biological significance has just begun to be revealed. In the present study, we have identified the PNGase ortholog from *Dictyostelium discoideum* using *in silico* analyses and describe its functional characterization. The major function of this protein is in degradation of misfolded glycosylated proteins; however we also show here its requirement during multicellular development.

### *D. discoideum* has a PNGase ortholog and shows structural homology to mouse PNGase

**Figure 1:**
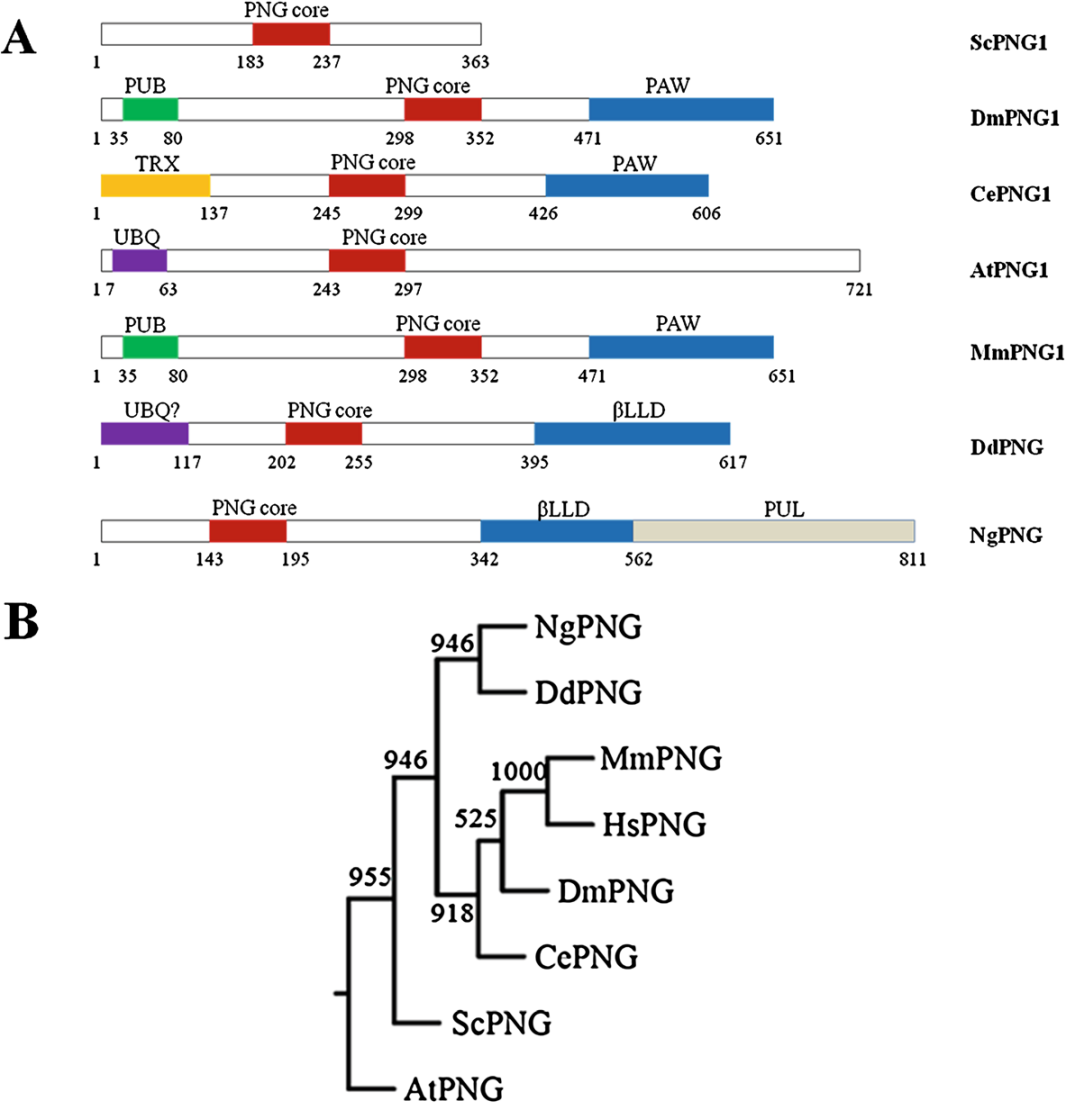
***Dd*PNGase shares sequence homology with known peptide: N-glycanase. **(**A**) Schematic representation of conserved domain architecture of PNGase orthologs from various species and the putative *Dd*PNGase. (**B**) Protein sequences described in (A) and that of human were used to create an unrooted phylogenetic neigbour- joining dendogram showing bootstrap values out of 1000. It was constructed using PHYLIP. *Dd*PNGase is grouped a significant number of times (946 out of 1000) with known *N. gruberi *PNGase. [*Homo sapiens, *HsPNGase (Q96IV0); *Drosophila melanogaster, *DmPNGase (Q7KRR5); *Saccharomyces cerevisiae, *ScPNGase (Q02890); *Caenorhabditis elegans, *CePNGase (Q9TW67); *Arabidopsis thaliana, *AtPNGase (Q9FGY9); *Dictyostelium discoideum, *DdPNGase (Q55FC8); *Naegleria gruberi, *NgPNGase (D2V850)].

The putative PNGase protein of *D. discoideum* shows maximum homology to an uncharacterized protein from another cellular slime mold, *Polyspondylium pallidum.* It also shows significant homology with PNGase core and β-lectin legume domains of PNGase of *Naegleria gruberi* (amoeba). PNGase core domains of *Phylophthora infestans* (fungus) and *Ostreococcus tauri* (green alga) also show high homology to the putative *Dd*PNGase*.* Domain architecture analyses of known PNGases (Figure 1A) show the presence of PNGase core domain. PNGases possess a carbohydrate binding domain (PAW) at the C-terminus which probably assists in binding to the glycan of the misfolded proteins. *Sc*PNGase lacks both N and C terminal domains and possess only a functional core domain. *Dd*PNGase possess a catalytic core domain (54 aa) and a C- terminal β-lectin legume (β-LL) domain (223 aa) for binding to glycan moiety. β-LL domain is originally present in lectins of legumes and requires tightly bound calcium and manganese ions for their interaction with carbohydrate. *Naegleria gruberi* PNGase also possess a highly homologous PNGase core and β-LL domains. The N-terminal region (1–117 aa) of putative *Dd*PNGase does not show high homology to any characterized protein at NCBI. BLASTP2 results at EMBL show its low homology (Score 36 bits, 31% identity, 58% positives) with UBQ (ubiquitin homolog) domain of ubiquitin carboxy terminal hydrolase of fungus *Neurospora crassa,* which is a de-ubiquitinating enzyme. It shows the presence of a deglycosylating core domain, glycan binding β-LL domain and probable de-ubiquitinating UBQ domain.

We observe diverse structural organization between species which may reflect on its diverse functions. For example, the *Ce*PNGase has a thioredoxin domain having an oxidoreductive activity apart from being a functional PNGase. Similarly, the PUB domain in *Drosophila* and mouse may also serve in protein-protein interactions.

The analysed PNGases were phylogenetically separated into three clades. *Dd*PNGase is closer to the PNGase of *Naegleria gruberi* (*Ng*PNGase). The putative *Dd*PNGase and *Ng*PNGase belong to one clade while the *Hs*PNGase, *Mm*PNGase, *Dm*PNGase and *Ce*PNGase grouped together in another clade. *Sc*PNGase and *At*PNGase are grouped together (Figure 1B).

**Figure 2:**
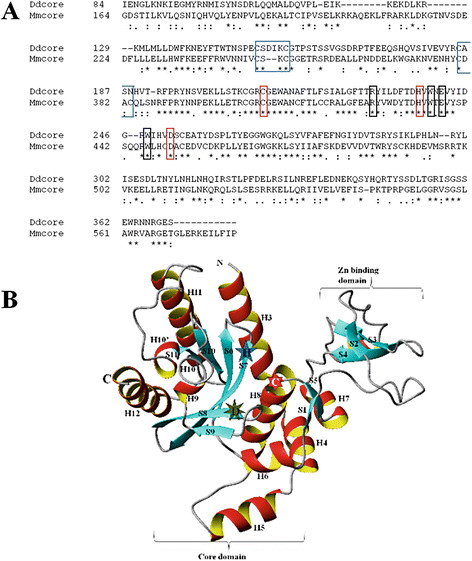
**Tertiary structure prediction of putative *Dd*PNGase.** (**A**) Multiple alignment of of the conserved core domain of *D. discoideum* and *M. musculus *is shown. The red coloured boxes show the conserved catalytic triad residues corresponding to C^210^, H^237^, and D^252^ from *D. discoideum*. The two CXXC motifs are shown in blue boxes. The conserved tryptophan (W239, W248), arginine (R229) and glutamate (E241) are shown in black boxes. The numbers in the left represent the position of the amino acid in the respective full length protein sequences. (**B**) Tertiary structure homology prediction model of *Dd*PNGase (core domain) based on mouse PNGase (2f4m-A) as a template was created by SWISS MODEL and viewed using MOLMOL.

Sequence alignment (Figure 2A) shows that *Dd*PNGase possess the catalytic triad residues Cys^210^, His^237^ and Asp^252^, corresponding to the conserved core residues of other PNGases. *Dd*PNGase also possess the corresponding tryptophan (W239 and W248), arginine (R229) and glutamate (E241) conserved residues which could possibly be essential for catalysis. Two CXXC motifs are present in *D. discoideum* but the second cystine of each motif is absent which probably may affect the Zn binding in this putative PNGase. The exact role of Zn binding is not understood but it has been suggested to modulate the enzyme tertiary structure in other systems. The cysteine residues of CXXC motif are essential for *S. cerevisiae* PNGase activity [3]. Studies on *C. elegans* N-glycanase (NGLY1) revealed that it contains two Zn binding motifs - C^191^PKC^194^ and C^225^DGC^228^, but it did not get activated by Zn binding. It suggests that all PNGase may not require Zn ion for their deglycosylation activity. In contrast to *Sc*PNGase, *Ce*PNGase activity was inhibited by Zn^2+^ and other metal ions such as Cu^2+^ and Co^2+ ^[27]. Although the mechanism of inhibition of PNGase activity by Zn^2+^ in *C. elegans* and activation in *S. cerevisiae* is not clearly understood but it may be due to changes in tertiary structure of the enzyme which could modulate the accessibility of the catalytic cleft to the substrate glycoprotein.

*Dd*PNGase is a single copy gene encoding a protein of 618 amino acids. In most cases, prediction of tertiary structure can validate the function of a novel protein. To model the 3D structure of an unknown protein, tertiary structures of the proteins which show significant sequence homology to the query protein are identified. These known structures are then used as a template for comparative homology modeling of the unknown protein tertiary structure. Prior to tertiary structure prediction, the 87–340 aa region around the Zn binding domain and core domain of *Dd*PNGase was submitted at the Jpred3 for secondary structure prediction. This confirmed the secondary structure around the core and Zn binding domains. We identified some known 3D structures of PNGases showing high homology to the primary sequence of our query protein (putative *Dd*PNGase) sequence while doing secondary structure prediction at Jpred3 server. The putative *Dd*PNGase primary sequence show maximum homology (41% identity, 55% positives) to the *M. musculus* PNGase core sequence. Its tertiary structure is available in the structural database as PDB entries 2f4m-A chain and 2f4o-A chain. PDB 2f4m is the tertiary structure of *Mm*PNGase complexed with HR23B and PDB 2f4o is *Mm*PNGase complexed with HR23B and a synthetic peptide inhibitor. High percent homology suggests that our query protein is probably a PNGase.

To create a tertiary structure of the *Dd*PNGase core region, mouse PNGase core tertiary structure (2f4m-A) was chosen as a template for homology modeling. Structural homologues of the three different domains of *Dd*PNGase, the N-terminal (1–84), core domain (85–376) and the C-terminal (377–618) depict a near perfect match with the template. Figure 2A shows the multiple alignment of the highly conserved region of the core domain from both *D. discoideum* and *Mus musculus.*

The core domain sequence of putative *Dd*PNGase was submitted to the SWISS MODEL (Version 8.05) server for modeling of tertiary structure based on alignment with the template protein (*Mm*PNGase core- 2f4mA). The model thus created by SWISS MODEL as viewed under the MOLMOL software is shown in Figure 2B. The *Dd*PNGase core region consists of six stranded antiparallel β-sheet (S6–S11) surrounded by eight α-helices (H3 to H9 and H11) on one side and four α-helices (H9, H10, H10’ and H12) on the other side of the sheet. The numbering of secondary structure elements has been done according to *Mm*PNGase tertiary structure as done by Zhao *et al *[22], with the N-terminal helix labeled as H3, as the first two helices of *Sc*PNGase are not present in the *M. musculus* and *D. discoideum* PNGases. The zinc binding domain contains a three-stranded β-sheet (S2–S4). A small β-sheet (S1 and S5) is located between the zinc binding and catalytic domains. Similar to *Mm*PNGase, C-terminal α-helices H11 and H12 of *Dd*PNGase may probably interact with Rad23. H5 α-helix and the loop following it are comparatively longer in *Mm*PNGase with 5 extra amino acids in each of them. H6 α-helix is half the size in *Dd*PNGase as compared to *Mm*PNGase (14 aa). A helical region of 5 amino acids in the Zn binding region between S2 and S3 as shown in the tertiary structure model of *Mm*PNGase (2f4mA) is also not mentioned in literature [28]. Even with the existing differences, we found that the cleft composed of H8 helix and six antiparallel β-strands to be completely superimposed. The position of catalytic triad residues is also conserved in both the PNGases. The α-helix H8 possess the catalytic Cys residue (*Dd* C^210^*Mm* C^306^), strand S7 possess catalytic His (*Dd* H^237^*Mm* H^333^) at the N-terminal end and strand S8 possess triad Asp residue (*Dd* D^252^*Mm* D^350^) at the C3-terminal end. Thus, the core domains of both the PNGases consist of six antiparallel β- strands (S6–S11) with helices around the sheet. The C-terminal H11 and H12 α-helices of mouse PNGase interact with the HR23 protein [29], and may be involved in the same interaction of *Dd*PNGase with HR23 or Rad23 homologue in *D. discoideum*. The stereochemistry of the model prepared for the core domain region of the putative *Dd*PNGase is good with 98% of the residues located in the most favoured and additionally allowed regions of the Ramachandran plot. 1% of the residues are located in generously allowed regions in the Ramachandran plot. Merely 1% residues could not be obtained in the allowed regions, as checked by Structure Analysis and VErification Server (SAVES) online resource. These residues are present in loop, bend and coil regions of the tertiary structure.

### Subcellular localization of *Dd*PNGase

**Figure 3:**
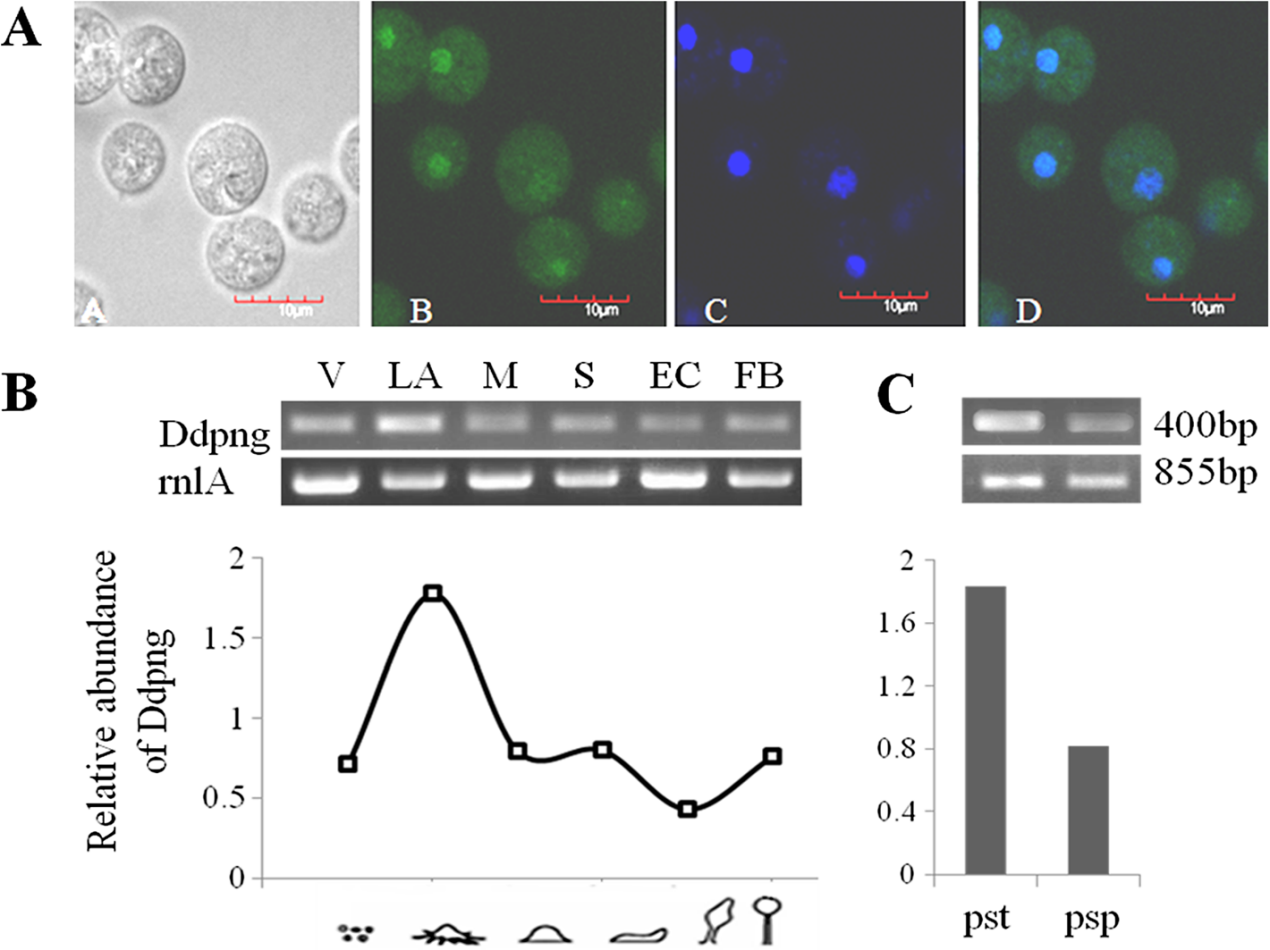
**Subcellular localization and RNA expression during development. **(**A**) Subcellular localization studies in constitutively expressing [*act15/png-eyfp*]/Ax2 cells. (A) DIC image; (B) DdPNGase-EYFP image; (C) DAPI stained image; (D) merged image. The fusion protein (green) is localized maximally in the nucleus (blue) and to a lesser extent in the cytosol. (**B**) Temporal expression pattern of *Dd*PNGase during development and differentiation. Graph representing the ratio of *Ddpngase *to *rnlA *expression levels at different time points of development. High transcript level was seen at t_6_ hours corresponding to loose aggregate stage. (**C**) Transcript levels and the graph showing the normalized expression levels of *Ddpngase *in isolated prestalk and prespore cells. [V-vegetative; LA-loose aggregate; M-mound; S-migrating slug; EC-early culminant; C-culminant; pst-prestalk; psp-prespore].

The Eyfp (green) tagged *Dd*PNGase from the *act15/png-eyfp*/Ax2] overexpressing strain colocalized in the nucleus along with DAPI blue stained DNA. It was also present to a lower extent in the cytosol (Figure 3A). The PNGases are generally localized in close proximity to the proteasomal machinery as they are proposed to participate in the proteasome dependent glycoprotein degradation pathway. The *S. cerevisiae* PNGase is reported mostly in the nucleus [16] where its proteasomes reside. In *D. discoideum,* the proteasomes are reported to be present in cytosol but in higher concentrations in the nucleus [30]. A part of it may also be localized on the ER membrane as in the case of mouse PNGase ortholog, Ngly1 [31] which was reported to be associated with the ER resident proteins such as gp78 [32] or Derlin [33].

### Expression levels and patterns of *Ddpngase* are developmentally regulated

Temporal expression pattern of the *Ddpngase* transcript during development was carried out by a semi- quantitative RT-PCR using intron spanning primers and was normalized against the constitutively expressed *rnlA* transcript. RNA was collected from different specific stages of development and also from isolated prestalk and prespore cells. Relative abundance of the transcript of *Ddpngase* with respect to the transcript of *rnlA* was plotted in Figure 3B. The RNA derived product with a predicted size of approximately 400 bp was amplified from all the developmental stages (from vegetative to fruiting body stages) taken but showed highest expression at the time of aggregation (both loose and tight aggregates appearing at t_6_ and t_8_ hours) and declined thereafter. There was slight increase at the fruiting body stage at t_24_ hours. There was more than 2.0 folds increased expression in the isolated prestalk cells as compared to the isolated prespore cells (Figure 3C). The absence of a 500 bp band that is expected from amplification of the genomic DNA confirmed the purity of the RNA (data not shown). These data confirm that *pngase* is transcribed throughout development.

**Figure 4:**
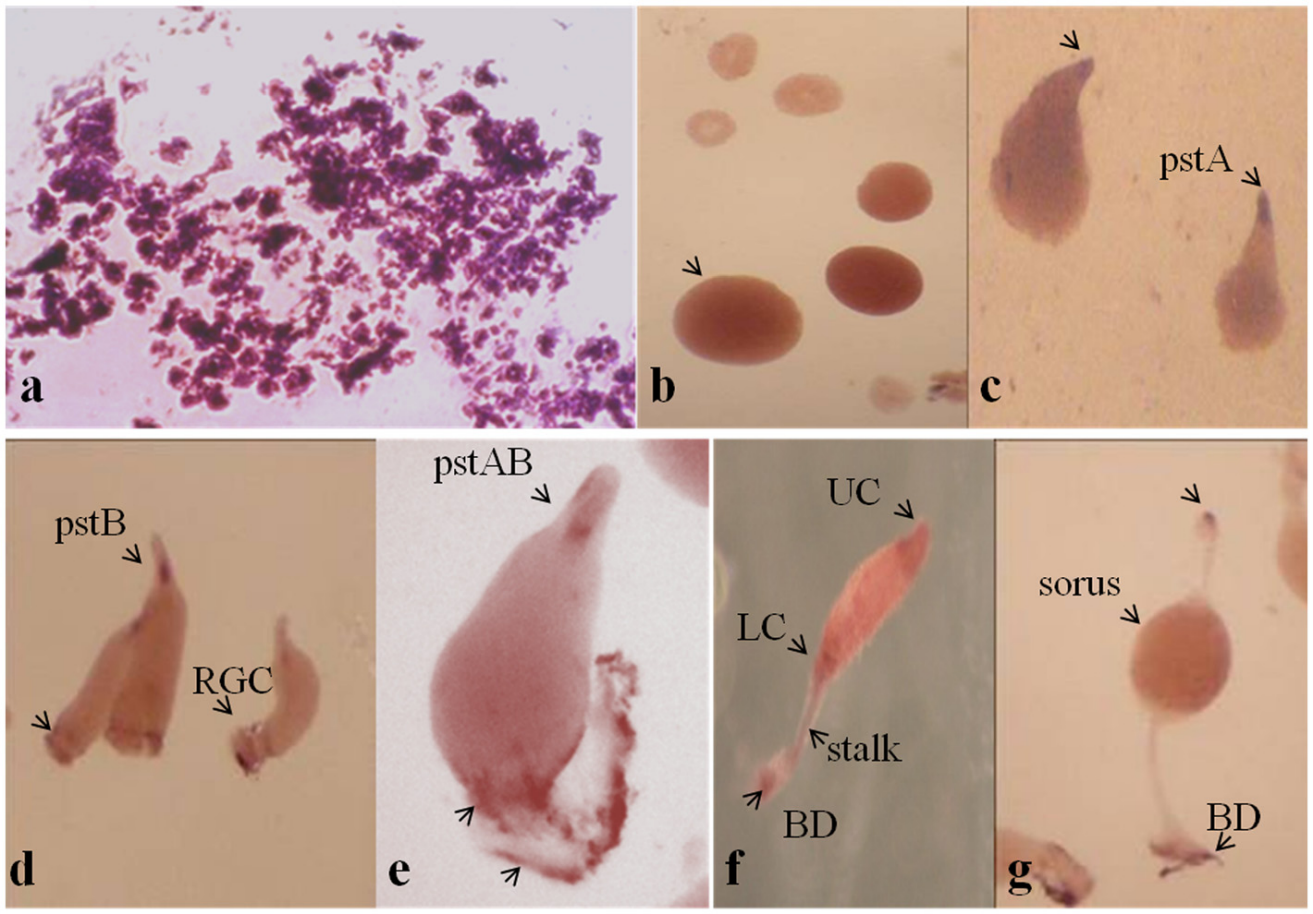
***In situ *hybridization by *Ddpngase *DIG labeled RNA probes. **The *in situ *hybridization with the *Ddpngase * antisense probe showed high expression during the loose (**a**) and the tight (**b**) aggregate stages. The expression was more concentrated in the tip (pstA) region (**c**) though posterior prespore region also showed faint expression. The expression of *Ddpngase* was highest in the prestalk B/AB cells and rear-guard cells (RGC) (**d** and **e**). The mid culminant (**f**) and the culminant (**g**) showed the expression in the upper (UC) and lower cups (LC) and the basal disc (BD) regions. The negative control *in situ *hybridization using sense RNA probes did not develop significant color or background staining (data not shown). The different regions in the multicellular structures needing particular attention are marked.

To examine the spatio- temporal expression pattern of *Ddpngase* during development, RNA *in situ* hybridization was carried in various multicellular stages of development (Figure 4). A strong hybridization signal was observed both in the loose (Figure 4a) and tight aggregates (Figure 4b) and by the first finger stage the expression got restricted largely to the prestalk region with very low levels in the prespore region (Figure 4c). By the time migrating slugs were formed, the expression further got restricted to the pstB cells in the prestalk region and the rear-guard cells (RGC) (Figure 4d). During early culmination the expression was found in the pstAB and RGC (Figure 4e). During mid-culmination (Figure 4f) the expression was seen in the upper and the lower cup region and also in the basal disc (BD). In the fruiting body stage (Figure 4g) the basal disc and the tip region showed higher expression while low expression was also seen in the sorus. PstA cells express extracellular matrix protein A (EcmA), pstB cells express extracellular matrix protein B (EcmB) and pstAB cells express both EcmA and EcmB proteins. The *ecmA* is strongly expressed in the anterior 10% of the slug (pstA region). The *ecmB* gene is expressed in the central core region of slug tip. The collar region which forms the border between the prestalk and prespore cells in the slug express low level of EcmA protein and are called as pstO cells. As culmination proceeds, pstA cells follow the pstB cells into the stalk tube and initiate the expression of *ecmB* gene and become pstAB cells. The pstAB cells form the basal disc along with the anterior-like cells (ALC). The ALC comprise approximately 15% of the posterior prespore zone. ALCs also migrate upward and downward to form part of upper and lower cup of the culminant. PstO cells also contribute to the upper cup formation.

The expression detected with *pngase* antisense probe is specific as no signal was detected using DIG labeled RNA probe prepared against the sense strand of cDNA. These results suggest that *pngase* transcript is present throughout development and they may play a critical role during the aggregate formation. Their presence also suggests their role in prestalk/stalk differentiation or patterning.

### Knockout mutants show defect in aggregation

To provide more insight into the biological significance of the PNGase ortholog in *D. discoideum* we generated and characterized both the overexpressing [*act15/png-eyfp*/Ax2] and knockout (*png*^*-*^/Ax2) mutant strains of the *pngase* gene. The knockout mutants were made by gene disruption followed by homologous recombination. The deletions were molecularly characterized by PCR with DNA primers flanking the deletion points and sequencing the resulting PCR products (data not shown). On the other hand, no transcripts could be detected from the knock out strains while the wild type and the Bsr integrant strain (RI) showed the transcript (data not shown). This result indicates that *png*^*-*^/Ax2 is a bonafide null allele of the *pngase*.

**Figure 5:**
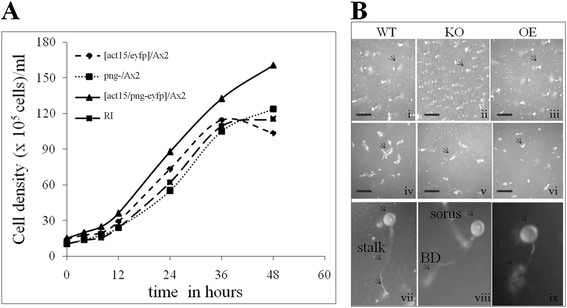
**Growth and development of deletion mutants of *Ddpngase *. **(**A**) Comparison of growth profiles of wild type ([*act15/eyfp*]/Ax2), knockout (*png*^*-*^/Ax2), overexpresser [*act15/png-eyfp*]/Ax2 and random integrant (RI) strains. Generation time (doubling time) was calculated in logarithmic phase cultures2.5-4x10^6^ cells/ml) using the formula: generation time = (t2-t1) x [log2/log (q2/q1)]. (**B**) Comparison of developments of wild type (WT), knockout (KO), and overexpressing (OE) strains are showed. Few developmental stages like mound stage (i, ii, iii); migrating slug stage (iv, v, vi) and fruiting body stage (vii, viii, ix) phenotypes are shown. Scale bar 1000 μm.

Having established mutant strains for *pngase* (both [*act15*/*png-eyfp*]/Ax2 and [*png*^*-*^/Ax2]), we then compared their growths and phenotypes with the wild type strain transformed with an empty vector ([*act15/eyfp*]/Ax2). Figure 5A shows the growth profiles of the wild type, knockouts, overexpressers and the random integrants (comparable to the wild type). The knockout mutant, [*png*^*-*^/Ax2], were found to be slow growers with a doubling time of 10.3± 0.6 hours, while the overexpressers, [*act15*/*png-eyfp*]/Ax2, were slightly fast growers with a doubling time 7.21 ± 0.34 hours as compared to the wild type and the random integrants which had a doubling time of 8.24 ± 0.41 hours.

The knockout strain, [*png*^-^/Ax2], developed slower, taking 2–3 hours more as compared to the wild type to form fruiting bodies (Figure 5B). They formed numerous small aggregates by approximately 12 hours of starvation as compared to the wild type which took almost 8 hours. The formation of the mound from the aggregate was faster as it took almost 2 hours while the wild type took almost 4 hours. Very few aggregates formed slugs which were medium sized. The transition from the migrating slug stage to the early culminant took only 2 hours as compared to the wild type which took 3–4 hours. The [*act15*/*png-eyfp*]/Ax2 cells showed normal development with timings similar to that of the wild type cells. Nevertheless, the size and morphology of different developmental stages formed were smaller than the wild type but appeared normal, though the number of aggregates culminating into fruiting bodies were lesser as compared to wild type cells. The fruiting bodies formed by the [*act15*/*png-eyfp*]/Ax2 cells were the smallest in size showing small sturdy stalks with enlarged basal disc and slightly larger sorus, while that formed by the [*png*^*-*^*/*Ax2] cells had slightly thinner stalk with sorus slightly bigger than that formed by the Ax2 cells but smaller than the knock out cells (Figure 5B vii–ix). As per our observations, many cells did not participate in development and very few aggregates were formed which finally culminated into fruiting bodies. From the observed transcript levels, one could speculate that the *pngase* may be required by the prestalk cells, and overexpression of this maybe biasing many cells towards the prestalk pathway as a result of which they die prematurely before they can take part in the development. We speculate that the lack of PNGase in *D. discoideum* probably inhibits the cell to cell signaling events responsible for the process of aggregation. There are chances that either cAMP signaling or cell polarity gets inhibited as a result of which the amoebae could not aggregate and only few such aggregates which could come in the near vicinity finally coalesce to form the multicellular structures. Transformation of the *png*^-^ cells with the plasmid containing the wild type PNGase expression driven under actin15 promoter could rescue the phenotype (data not shown).

Loss of function mutations has been carried out in different systems like *Arabidopsis *[34], *C. elegans *[17] and yeast. The plant did not show any change in its phenotype as well as in growth and development but it was found to limit axon branching during organ formation in *C. elegans*. Loss of function in *D. discoideum* brought about changes in growth, development as well as cell differentiation. Its loss slowed down the growth of the cells by nearly 2 hrs. Our observations suggest that PNGase plays a role in regulating the number of aggregates which would develop to form multicellular structures. The *png*^-^ cells do make small aggregates upon starvation but only few of them proceeded with further development. This suggests that PNGase may be involved in cell to cell signaling required for aggregation with a possibility of defects in cAMP signaling or in cell polarity as seen in case of *Neurospora *[18]. Since the *png*^-^ cells later went on to develop normal sized fruiting body with slightly longer stalks, one could say that PNGase may be required for the signaling taking place during aggregation whose consequences are required for cell type differentiation mainly of the prestalk cells.

### *Dd*PNGase is a functional deglycosylating enzyme and does not show transglutaminase activity

**Figure 6:**
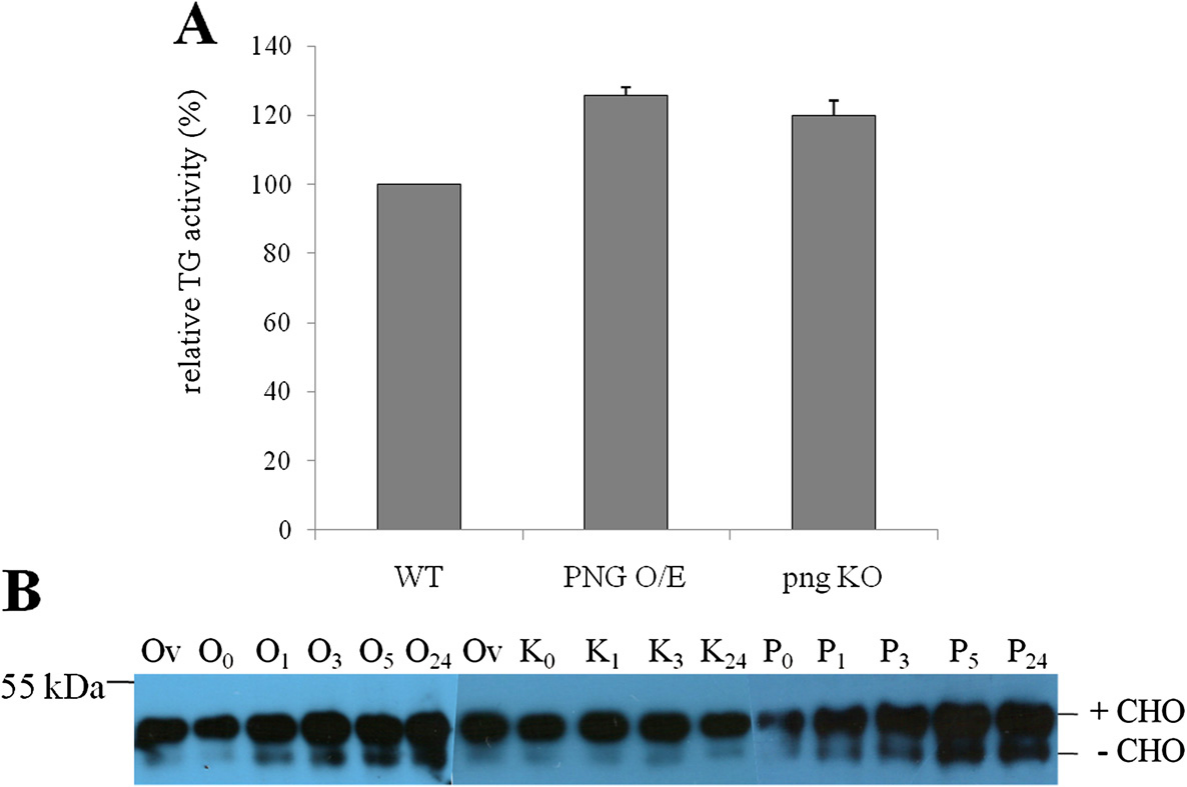
**Transglutaminase and deglycosylation activity measurements in various strains developed.**(**A**) TG activity was measured in wild type, Ax2 (WT), overexpresser, [*act15/png-eyfp*]/Ax2] (PNG O/E) and knockout, *png*^*-*^/Ax2 (pngKO) cells. Activity in WT was taken as 100%. No significant decrease in activity in the pngKO cells was observed. (**B**) Deglycosylation activity using denatured ovalbumin as a substrate was measured using commercially available pure PNGase F and lysates of [*act15/png-eyfp*]/Ax-2 and (png^-^/Ax2) cells at various time points (0 h, 1 h, 3 h, 5 h and 24 h) by Western blot analysis. The glycosylated (+CHO) band was seen in all the lanes and was comparable to that observed in lane 1 and 7 where ovalbumin was incubated without any enzyme for overnight. When purified enzyme (lanes 12–16) and lysate from PNGase overexpressing strain was used, a time dependent increase in the lower deglycosylated (−CHO) band was observed (lanes 2–6). The absence of this -CHO band shows the loss of deglycosylation activity in the knock out strain (lanes 8–11). +CHO and -CHO indicate glycosylated and deglycosylated forms of ovalbumin respectively. [Ov-ovalbumin; P-purified PNGase F; O-[*act15/png-eyfp*]/Ax-2; K- (png^-^/Ax2); 0–0 h reaction;1-1 h reaction; 3–3 h reaction; 5-5 h reaction; 24-24 h reaction].

Our search for the gene having transglutaminase activity in *D. discoideum* resulted in the identification of few transglutaminase-like superfamily members, of which one of them was identified as a peptide: *N*-glycanase during further *in silico* analyses of the protein sequence. We found a small but statistically insignificant increase in TG activity in the *act15*/*png-eyfp*/Ax2 cells over the wild type Ax2 cells (Figure 6A). The *png* null strain did not show any reduction in the TG activity suggesting that *Dd*PNGase does not have TG activity. Della Mea and group [35] had also found transglutaminase activity in the recombinant *At*PNGase1 but could not detect its loss in the knockout strain.

We analyzed the capability of *Dd*PNGase to deglycosylate the native and denatured ovalbumin substrates. Crude extracts of [*act15*/*png-eyfp*]/Ax2 and [*png*^*-*^*/*Ax2] strains, were incubated with either native or denatured ovalbumin in appropriate buffer conditions and the reaction was stopped at varying time points. The activity was compared with that obtained with the commercially available purified PNGase F (Figure 6B). In each case, a glycosylated (+CHO) band comparable to that seen with ovalbumin (Ov) after overnight incubation without the enzyme was observed. When pure enzyme was used, we saw a time dependent increase in the intensity of the lower deglycosylated band (−CHO) (Figure 6B, lanes 12–16). When we measured the deglycosylating activity present in the lysate of the *Dd*PNGase overexpressing strain, we found similar results showing a time dependent increase in the intensity of the -CHO band (Figure 6B lanes 2–6). A time dependent study using the knockout strain showed the lower deglycosylated band (Figure 6B, lanes 8–11), comparable to that observed with only ovalbumin (Figure 6B, lane 7) confirming the loss of PNGase activity in them.

Figure 6B shows the results obtained using denatured ovalbumin as substrate. The native ovalbumin did not show any deglycosylation activity and thus are not shown here. Joshi *et al*[25] had utilized the two glycoprotein substrates namely, yeast carboxypeptidase and chicken egg albumin (ovalbumin) to study the deglycosylation activity of yeast PNGase and its mutants and showed that misfolding of the glycoproteins was a prerequisite for PNGase mediated deglycosylation. Funakoshi *et al*[13] have provided evidence of an essential deglycosylation independent activity for *Dm*PNGase. A mutation in the gene led to the failure in proper multicellular development demonstrating the functional importance of the PNGase apart from its deglycosylation activity in *D. discoideum*. What is interesting is that the modulation of the level of PNGase in *D. discoideum* did not affect drastically the overall developmental patterns even when the transcript levels showed cell type biasness.

## Conclusions

In summary, we have identified and characterized a novel PNGase from *D. discoideum.* The *Dd*PNGase is a functional peptide:N-glycanase enzyme possessing deglycosylation activity, but does not possess transamidation activity. *Dd*PNGase was identified based on the presence of a common TG core domain and its sequence homology with the known PNGases. The domain architecture and the sequence comparison validated the presence of probable functional domains in *Dd*PNGase. The tertiary structure matched with the mouse PNGase the most. RT-PCR data revealed almost 3.0 folds increase in *pngase* transcript during aggregation stage after which they decline suggesting its importance during the process of aggregation. The *pngase* transcript is abundant in the prestalk regions, specifically in the pstB and pstAB regions. This needs to be further studied to find out the role in differentiation. PNGase showed nuclear localization. Unlike yeast, mutation in the *Dd*PNGase caused developmental aberrations indicating its importance in multicellular development. Apart from having deglycosylation activity and a role in development it requires further characterization to find its precise role in differentiation.

## Competing interests

There is no conflict of interest with the authors.

## Authors’ contributions

AG and RL contributed equally to the work. The concept and manuscript was mostly written by SS and partly by AS. The manuscript in the present form has the consent of all the four authors. All authors read and approved the final manuscript.
